# The rise of multiple imputation: a review of the reporting and implementation of the method in medical research

**DOI:** 10.1186/s12874-015-0022-1

**Published:** 2015-04-07

**Authors:** Panteha Hayati Rezvan, Katherine J Lee, Julie A Simpson

**Affiliations:** 1 grid.1008.9000000012179088XCentre for Epidemiology and Biostatistics, Melbourne School of Population and Global Health, The University of Melbourne, Parkville, Melbourne, VIC Australia; 2 grid.1058.c000000009442535XClinical Epidemiology and Biostatistics Unit, Murdoch Childrens Research Institute, Parkville, Melbourne, VIC Australia; 3 grid.1008.9000000012179088XDepartment of Paediatrics, The University of Melbourne, Parkville, Melbourne, VIC Australia

**Keywords:** Missing data, Multiple imputation, Sensitivity analysis, Reporting

## Abstract

**Background:**

Missing data are common in medical research, which can lead to a loss in statistical power and potentially biased results if not handled appropriately. Multiple imputation (MI) is a statistical method, widely adopted in practice, for dealing with missing data. Many academic journals now emphasise the importance of reporting information regarding missing data and proposed guidelines for documenting the application of MI have been published. This review evaluated the reporting of missing data, the application of MI including the details provided regarding the imputation model, and the frequency of sensitivity analyses within the MI framework in medical research articles.

**Methods:**

A systematic review of articles published in the *Lancet* and *New England Journal of Medicine* between January 2008 and December 2013 in which MI was implemented was carried out.

**Results:**

We identified 103 papers that used MI, with the number of papers increasing from 11 in 2008 to 26 in 2013. Nearly half of the papers specified the proportion of complete cases or the proportion with missing data by each variable. In the majority of the articles (86%) the imputed variables were specified. Of the 38 papers (37%) that stated the method of imputation, 20 used chained equations, 8 used multivariate normal imputation, and 10 used alternative methods. Very few articles (9%) detailed how they handled non-normally distributed variables during imputation. Thirty-nine papers (38%) stated the variables included in the imputation model. Less than half of the papers (46%) reported the number of imputations, and only two papers compared the distribution of imputed and observed data. Sixty-six papers presented the results from MI as a secondary analysis. Only three articles carried out a sensitivity analysis following MI to assess departures from the missing at random assumption, with details of the sensitivity analyses only provided by one article.

**Conclusions:**

This review outlined deficiencies in the documenting of missing data and the details provided about imputation. Furthermore, only a few articles performed sensitivity analyses following MI even though this is strongly recommended in guidelines. Authors are encouraged to follow the available guidelines and provide information on missing data and the imputation process.

**Electronic supplementary material:**

The online version of this article (doi:10.1186/s12874-015-0022-1) contains supplementary material, which is available to authorized users.

## Background

Missing data are common in clinical and epidemiological studies [1-3], and may arise for a variety of reasons that are beyond the researcher’s control. Sometimes an individual refuses to participate in a wave of data collection (‘unit non-response’), or does not provide information about a particular measure (‘item non-response’). Statistical analyses that exclude individuals with missing data result in estimates with less precision compared with the analysis of all individuals, and more importantly, may lead to biased inference. Applying statistical methods that handle missing data appropriately may reduce the bias and increase the precision of the estimates obtained [3]. One of the main difficulties in the analysis of incomplete data is determining the most appropriate approach to handle the missing data. Several statistical methods have been proposed in the literature for handling missing data [4]. These include the simple approach of excluding all individuals with missing data (termed a complete case analysis (CC)), single imputation methods such as last observation carried forward (LOCF), and more principled methods such as multiple imputation (MI). Each of these approaches makes assumptions regarding the missing data that cannot be verified from the observed data. Thus, researchers are encouraged to carry out a sensitivity analysis to assess the robustness of the results to plausible departures from the missing data assumption made in the main analysis [5-8].

A review of published randomised controlled trials in *JAMA*, *New England Journal of Medicine*, *BMJ*, and the *Lancet* from July to December 2001, by Wood et al. [3], observed that the majority of papers chose an inadequate approach for handling missing outcome data in the analysis of randomised controlled trials, and that sensitivity analyses were rarely performed to justify the assumptions made in the main analysis. This review also provided recommendations for avoiding and handling missing data. These recommendations included reporting any descriptions of missing data (e.g. proportion of missingness in each treatment arm, difference between key baseline variables between individuals with observed and missing outcomes, etc.), stating the missing data assumptions for the statistical approach selected for handling the missing data, and performing sensitivity analyses around the method chosen to handle the missing data when there is a large proportion of missing data. A more recent review of published clinical trials (2005–2012) with missing longitudinal outcomes by Powney et al. [1] reported ongoing inadequacies in the reporting and handling of missing data. They found that the majority of the published trials did not provide reasons for missingness (drop out) or justification for the methods used to handle missing data. In line with Wood et al. and Powney et al., the Strengthening the Reporting of Observational Studies in Epidemiology (STROBE) statement published in 2007 [9] recommends that the number of individuals with missing data for each variable of interest should be specified as well as possible reasons for non-participation or non-response at each stage of the study. STROBE also states that the method for handling missing data should be detailed in the statistical methods. A review of publications from cohort studies with exposures measured in multiple follow-up waves by Karahalios et al. [2], points out the continuing use of inappropriate methods of missing data handling and the lack of adherence to the STROBE guidelines. Finally, a report from the National Research Council (NRC) [8], published in 2010, states the necessity of conducting sensitivity analyses using different approaches to deal with missing data to explore the robustness of conclusions to alternative assumptions about the missing data.

Over recent years, multiple imputation (MI) has gained popularity as a powerful statistical tool for handling missing data [10] and is starting to be recommended by journal reviewers [7]. Although MI is appealing and fairly easy to implement in standard statistical software, it can introduce bias if not carried out appropriately [11]. Despite the growth in popularity of MI, a review by Mackinnon [10] highlighted that there was inconsistent reporting of MI in research articles published in major medical journals (i.e. *JAMA*, *New England Journal of Medicine*, *BMJ*, and the *Lancet*) from the earliest date of full text searching for MI until the end of 2008. Since then, Sterne et al. [5] suggested guidelines for the conduct and reporting of analyses using MI, in which authors are encouraged to document the important aspects of the implementation of the MI procedure in order to assist readers to make informed decisions about the analysis performed.

More recently, it is also starting to become apparent that even when MI is carried out, it may be important to conduct a sensitivity analysis regarding the (untestable) missing at random assumption around MI, since it also makes fairly restrictive assumptions about the missingness [8]. However, it is unclear whether such sensitivity analyses are being carried out after performing MI.

Previous reviews of missing data have generally focused on documenting the handling and reporting of missing outcome data in randomised clinical trials or missing covariate measures in cohort studies with multiple waves of data collection. In this systematic review, our focus is more specifically on assessing the implementation and documentation of MI in both published randomised control trials and observational studies, in addition to the reporting of missing data when using MI. Although we recognise the importance of correct specification of the imputation model in order to provide valid results of MI, we focus on whether a detailed description of variables included in the imputation model and information on the imputation method are provided, rather than judging the adequacy of the imputation model.

Our systematic review extends the review proposed by Mackinnon [10] assessing whether the reporting of MI has improved over recent years as the popularity of this approach has increased and guidance has appeared in the literature. Furthermore, this review explores whether researchers are recognising the assumptions made when using MI and are conducting sensitivity analyses within the MI framework to assess the impact of this assumption. To do this, we report a detailed exploration of how MI was carried out and reported within both trials and observational studies that use MI in two high ranking medical journals, the *Lancet* and the *New England Journal of Medicine*, from 2008 through 2013.

The rest of this paper is set out as follows: we begin with an overview of the steps involved in performing MI to highlight the detail of reporting required. This is followed by an explanation of the inclusion criteria and extraction details for the systematic review. Finally, we summarise the results of the review, and then end with a discussion and draw conclusions.

## Methods

### Implementation of multiple imputation

MI is a sophisticated but flexible approach for handling missing data and is broadly applicable within a range of standard statistical software packages such as R [12], SAS [13] and Stata [14]. MI proceeds with replicating the incomplete dataset multiple times and replacing the missing data in each replicate with plausible values drawn from an imputation model. The statistical analysis of interest (e.g. a logistic regression of binary outcome on an exposure variable and confounders) is then performed on each completed dataset separately. Finally, a single MI estimate (and its standard error) is calculated by combining the estimates (and standard errors) obtained from each completed dataset using ‘Rubin’s rules’ [15,16]. Unlike single imputed methods, MI takes into account the uncertainty associated with the imputed values. The estimated variance of the overall MI estimate allows for within-imputation (i.e. the uncertainty in the estimate within each completed dataset) and between- imputation (i.e. the uncertainty between the estimates across the completed datasets) variability [15,16].

There are a number of decisions to be made in the imputation stage of MI which can affect the results obtained. It is important to document these decisions so that the readers can be clear about how the investigator performed the imputation and assess the validity of the results. For example, which variables should be included in the imputation model, what imputation method should be used, and how many imputations are required. Once these decisions have been made, it is important to perform diagnostic checks in order to assess the adequacy of the resulting imputation model.

Careful attention is required to select which variables should be included in the imputation model and the form of the variables in order to avoid misspecification of the imputation model and produce biased results. It has been widely recommended to include all variables used in the analysis model (including the outcome variable and any interactions or non-linear terms) in the imputation model to ensure congeniality between the imputation and analysis models. It is also important to include auxiliary variables (i.e. those variables that are not included in the analysis model but are potential predictors of missingness and/or the variable with missing data) in the imputation model which can be used to improve the accuracy of the imputed values [17,18].

Another important decision is the method of imputation. There are several methods in the statistical literature for performing MI. In univariate imputation, where only a single variable has missing data, the imputation model can be tailored to the variable being imputed, for example, a linear regression model for a continuous variable or a logistic regression model for a binary variable. Predictive mean matching (PMM) [19] (where missing data are imputed using the observed values with the closest predictive mean from a linear regression model) is another univariate method for imputing missing data for continuous variables, and is less sensitive to violation of the normality assumption than standard linear regression imputation. In multivariate imputation, where multiple variables have missing data, there are two approaches that are available in standard statistical software. These are MI by chained equations (MICE) (also known as fully conditional specification (FCS)) [20] where separate, conditional univariate imputation models are specified for each variable with missing data, and multivariate normal imputation (MVNI) [21] which uses a joint normal distribution applied to all of the variables with missing data. Both of these approaches are used in practice and it is currently unclear which approach is preferable [21].

With regards to the number of imputations that should be performed, it has been suggested recently to apply a reasonable number of imputations (>5) to avoid producing a large Monte Carlo error [20,22,23]. White et al. [20] argued that the number of imputations should be at least greater than the percentage of the missing data in the analysis (e.g. for 30% missing data at least 30 imputations should be performed).

The standard application of MI assumes that data are ‘Missing at random’ (MAR), meaning that the probability of data being missing depends on the observed data but not the missing data. When the underlying missing data mechanism is ‘Missing completely at random’ (MCAR), that is, the probability of missingness is independent of the observed and missing data, MI in most scenarios will be more efficient than CC analysis [24]. Recent literature surrounding MI recommends performing a sensitivity analysis following MI to assess for departures from the MAR assumption since in practice there is no way to identify the real missing data mechanism. The idea of a sensitivity analysis is to specify a range of possible values which measure the departure from MAR and assess the impact of these departures on the MI results. The weighting approach (a selection based method) [25,26], and the pattern-mixture approach [25,27] are two methods that have been proposed in the literature for conducting sensitivity analyses within the MI framework. These methods are not readily accessible in most of the statistical software packages at present; however, SAS has recently extended the MI procedure for conducting sensitivity analyses to the MAR assumption using the pattern-mixture model approach [28]. It is currently unclear how often such sensitivity analyses are conducted.

### Inclusion criteria and extraction details for this review

To explore the conduct and reporting of MI in the current medical literature, we reviewed research articles that were published between January 2008 and December 2013 in the *Lancet* and *New England Journal of Medicine (NEJM)* in which MI was implemented. These two leading medical journals were chosen since they were both included in the original review by Mackinnon [10], and their impact factor (i.e. the average number of citations of articles from a published academic journal) over the last six years (2008–2013) remains very high [29]. Additionally, the number of extracted articles that used MI during this period in these two journals was 103, similar to the number of articles reviewed in previously published reviews of reporting and handling of missing data [1-3,10,30,31].

The articles were identified using full-text search for the term “multiple imputation” in each journal’s website. There were no restrictions placed on the number of articles or the study design. Any supplementary materials or web appendices provided by the publisher were included in the review. The articles were all extracted and reviewed by one researcher (*PHR*). For any papers with uncertainty or ambiguity in the information regarding missing data or imputation process, the information was extracted by another researcher (*JAS*; ~25% of articles), and if required, any discrepancies resolved with a third researcher (*KJL*). For our systematic review, we followed the PRISMA guidelines for transparent reporting of systematic reviews (see Additional file 1: Table S1).

The articles were classified based on the type of the study design: (1) trials including randomised-controlled trials (RCTs) and non-randomised controlled trials (e.g. Quasi-experimental studies), and (2) observational studies including prospective, retrospective, cross-sectional and case–control studies (see Table 1). The following data were extracted from each study (where possible): the amount of missing data (i.e. proportion of observations with missing data or the proportion of complete cases), details on the MI procedure (including the type of variable(s) imputed, the number of imputed variable(s), the imputation method, the number of imputations, the variables included in the imputation model, the transformations applied to improve the normality of continuous variables, the imputation software used, whether diagnostic checks were employed to assess the imputation model, and whether MI was conducted as a primary or secondary analysis) and whether there were any sensitivity analyses conducted following MI.

**Table 1:** **Number of articles using multiple imputation from January 2008 to December 2013 by type of study**

	**Journal’s name**		
**Type of studies**	**Lancet**	**New England Journal of Medicine**	**All Studies**
	**(n = 58)**	**(n = 45)**	**(n = 103)**
**Trials**			
Randomised controlled trials	35	34	69
Non-randomised controlled trials^a^	2	2	4
Total	37	36	73
**Observational studies**			
Prospective studies	11	7	18
Retrospective studies^b^	6	1	7
Cross-sectional studies	3	0	3
Case–control studies	1	1	2
Total	21	9	30

^a^Quasi- experimental studies; ^b^Retrospective studies- these are studies which performed a retrospective analysis of routinely collected data.

## Results

**Figure 1:**
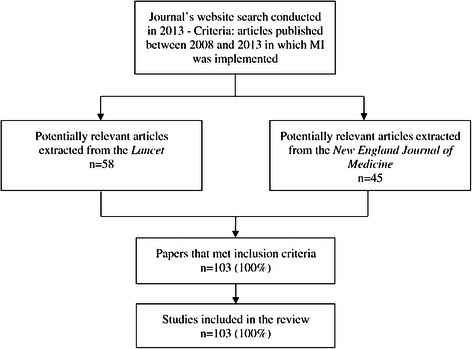
**Search results.**

Overall, we identified 103 published articles using MI during the study period (Figure 1) [32-134]. Of these articles, 73 (71%) were trials (see Table 1 and Additional file 2: Table S2).

**Figure 2:**
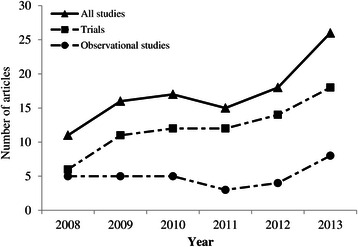
**Number of articles in the Lancet and New England Journal of Medicine that used MI: overall and by study type.**

Figure 2 shows an increasing trend over time (from 2008 to 2013) in the number of articles using MI. This trend appears to be more visible in trials than observational studies. The key findings of this systematic review including the details of reporting of missing data, variables imputed, and MI procedure are summarised in Tables 2, 3 and 4, respectively

**Table 2:** **Reporting of missing data in articles using multiple imputation**

	**Type of studies**		
**Characteristics reported**	**Trials**	**Observational studies**	**All studies**
	N (%)*	N (%)*	N (%)*
	**(n = 73)**	**(n = 30)**	**(n = 103)**
**Missingness**			
Availability of any information about the amount of missing data/complete cases	46 (63)	23 (77)	69 (67)
Proportion of complete cases was stated/available	31 (42)	10 (33)	41 (40)
Median [Range] % complete cases	88 [47-99]	41 [28-74]	84 [28-99]
Proportion of missing by each variable was stated/available	36 (49)	19 (63)	55 (53)
Assessed differences between individuals with complete and incomplete data in text?	8 (11)	5 (17)	13 (13)
Provided a table	3	4	7
Statement regarding missing data mechanism assumed in the analysis	13 (18)	7 (23)	20 (19)

*Unless otherwise stated.

**Table 3:** **Reporting of variables imputed in articles using multiple imputation**

	**Type of studies**		
**Characteristics reported**	**Trials**	**Observational studies**	**All studies**
	N (%)*	N (%)*	N (%)*
	**(n = 73)**	**(n = 30)**	**(n = 103)**
**Variables imputed**			
Variables imputed specified/available	61 (84)	28 (93)	89 (86)
Number of variable(s) imputed			
1	31	8	39
2	9	7	16
>2	17	10	27
Unclear^a^	4	3	7
Outcome variable imputed			
Yes	55 (75)	9 (30)	64 (62)
Not stated	12 (16)	2 (7)	14 (14)
No	6 (8)	19 (63)	25 (24)
Type of outcome variable imputed			
Numerical	31	6	37
Categorical	16	2	18
Numerical and categorical	8	1	9
Number of imputed outcome variables			
1	30	6	36
2	10	1	11
>2^b^	12	2	14
Unclear^a^	3	0	3
Covariate imputed			
Yes	13 (18)	21 (70)	34 (33)
Not stated	12 (16)	2 (7)	14 (14)
No	48 (66)	7 (23)	55 (53)
Type of covariates imputed			
Numerical	6	4	10
Categorical	3	8	11
Numerical and categorical	3	8	11
Unclear^a,c^	1	1	2
Number of imputed covariates			
1	6	3	9
2	2	7	9
>2	5	8	13
Unclear^a^	1	3	4

*Unless otherwise stated.

^a^Authors provided a generic statement regarding the imputed variables (e.g. the missing data in the covariates were imputed), and did not explicitly specify which outcome or covariate with missing data was imputed, so the number or type of imputed variables could not be verified.

^b^One article [128] imputed missing data in 5 incomplete variables for two questionnaires recorded at 6 different waves of data collection (i.e. 60 imputed variables).

^c^In one paper [98], the use of MI for imputing missing data in the covariates was derived from the cited reference, so the data type of imputed variables was not clear.

**Table 4:** **Reporting of MI procedure in articles using multiple imputation**

	**Type of studies**		
**Characteristics reported**	**Trials**	**Observational studies**	**All studies**
	N (%)*	N (%)*	N (%)*
	**(n = 73)**	**(n = 30)**	**(n = 103)**
**Imputation details**			
Any imputation details provided^a^	60 (82)	27 (90)	87 (85)
Imputation method stated	29 (40)	9 (30)	38 (37)
MI using chained equations (MICE)	14	6	20
MI using multivariate normal model (MVNI)^b^	7	1	8
MI using predictive mean matching (PMM)	1	0	1
MI using regression-based imputation^c^	4	1	5
MI using MICE & PMM^d^	1	1	2
MI using propensity score	1	0	1
MI using propensity score or regression modelling^e^	1	0	1
General procedure/command specified	5 (7)	2 (7)	7 (7)
Proc MI	4	1	5
MI command	0	1	1
Model-based MI^f^	1	0	1
Imputation method inferred	11 (15)	10 (33)	21 (20)
MICE (SAS- IVEware)	1	2	3
MICE (Stata- pre V11)	1	2	3
MICE (Multiple package^g^)	1	0	1
MVNI (SAS- pre V9.3-imputed more than 1 variable)	5	1	6
MVNI (R-Amelia II)	0	2	2
MVNI (S-plus)	2	0	2
Regression-based imputation (SAS pre V9.3-imputed 1 categorical variable)	1	3	4
Non-normal variables transformed prior to imputation	6 (8)	6 (20)	12 (12)
Log transformation^h^	4	4	8
Logit transformation	0	1	1
General comment about applying normalising transformation	2	1	3
Provided details on the variables included in the imputation model	26 (36)	13 (43)	39 (38)
Included auxiliary variable(s)	6	4	10
Included interaction term(s)	2	2	4
Included auxiliary variable and interaction	3	2	5
No information provided on auxiliary variables and interaction terms	15	5	20
Number of imputations	28 (38)	19 (63)	47 (46)
≤5	8	3	11
10	6	3	9
11-50	8	6	14
100	4	6	10
>100	2	1	3
Carried out diagnostic checks of the imputation model^i^	0 (0)	2 (7)	2 (2)
Assessed differences between results obtained from CC/LOCF and MI in the text/table^j^	45 (62)	17 (57)	62 (60)
**Software details**			
Imputation software stated^k,l^	51 (70)	25 (83)	76 (74)
SAS	23	10	33
Stata	18	9	27
R	6	6	12
Other packages (SOLAS, S-plus, SPSS)	4	0	4
**Analysis status of MI**			
MI used in the primary analysis	26 (36)	12 (40)	38 (37)
MI used as a secondary analysis	47 (64)	19 (63)	66^l^ (64)
Methods used for primary analysis if MI applied as a secondary analysis			
Complete case analysis (CC)^m,n^	43	19	62
Last observation carried forward (LOCF)	4	0	4
Sensitivity analysis following MI	3 (4)	0 (0)	3 (3)
Pattern-mixture model approach	1	0	1
Selection model approach	0	0	0
Performed but the method not stated^o^	2	0	2

*Unless otherwise stated.

Abbreviations: MI- multiple imputation, MICE- multiple imputation by chained equations, MVNI- multivariate normal imputation, PMM- predictive mean matching, MCMC- Markov chain Monte Carlo, CC- complete case, LOCF- last observation carried forward.

^a^Any information provided by the authors with regard to the imputation process. Note: a general procedure/command stated by the authors, and the imputation methods that were inferred by the reviewers are not included in this category.

^b^In five articles [35,61,68,90] MI via MCMC algorithm was used for imputing missing data.

^c^In three articles [40,47,84], logistic regression method and in two articles [39,113], linear regression method were stated as a imputation method for handling missing data.

^d^Two articles [61,93] imputed one or two variables with missing data under PMM (because of non-normality), and imputed other incomplete variables under MICE.

^e^One article [91] stated that MI was used on the basis of either propensity scoring or regression modelling for imputation of missing data in the primary and secondary outcome measures.

^f^One article [51] stated that model-based MI was used to account for missing data in the clinical outcome.

^g^In one article [77] multiple packages were used for the analyses, i.e. SPSS version 15.0 and Stata version 10.1. The default imputation method in either of these packages (given the specified versions) was chained equations.

^h^One article [93] used both the square root and log transformations for non-normally distributed variables.

^i^Both articles [82,130] compared the observed and imputed data.

^j^The MI estimates were not provided in 6 articles [34,37,81,85,87,120], instead a comparison of the results between the different approaches for dealing with the missing data was commented on in the text (e.g. the analysis of complete cases and the imputed data provided the same results).

^k^For eight articles [59,77,81,88,94,96,115,127] it was not possible to extract this information because multiple packages for the statistical analyses were mentioned with no explicit statement regarding which package was used for imputation.

^l^Those articles that did not provide the name of the imputation software (R, Stata, SAS, etc.), but instead gave the name of the procedure/application used for imputing missing data (e.g. Amelia II, IVEware) were also included here.

^m^One article [99] used MI as well as CC for primary analysis to impute the missing confounder values (with no imputation of missing data in the exposure and outcome), and used MI again as a sensitivity analysis to impute missing data in all confounders and the outcome (but not the exposure), as well as a CC.

^n^Two articles [40,100] used LOCF for the secondary analysis as well as MI; one of them described the MI as a sensitivity analysis.

^o^A general statement was made about performing a sensitivity analysis but the results of the details were not provided.

### Reporting of missing data

The characteristics reported regarding the missing data are summarised in Table 2. Overall, 69 papers (67%) provided some information about the amount of missing data; this includes 46 out of 73 trials (63%) and 23 out of 30 observations studies (77%). Of the 69 with information about the amount of missing data, the proportion of complete cases was presented or possible to infer in 41 papers (59%), and the amount of missing data by variable was reported or inferable in 55 papers (80%). In those with data available on the proportion of complete cases, this ranged from 28% to 99%, with a median of 84%. The maximum proportion of missing data for a single variable was 72%.

Very few articles (n = 13, 13%) compared the distribution of participant characteristics (e.g. exposure, outcome, demographic variables) between individuals with complete and incomplete data; of these, 7 provided summary tables. Out of 103 reviewed papers, only 20 studies (19%) provided an explicit statement regarding the missing data assumption used in the primary analysis (e.g. MCAR or MAR).

### Variables imputed

The characteristics of imputed variables reported in the identified articles are summarised in Table 3. In 89 articles (86%), imputed variables were directly specified or possible to identify from the text. The number of imputed variables varied from 1 to 60, with the majority (n = 39) imputing missing data in a single variable, 16 imputing missing data for two variables and 27 imputing missing data for more than two variables. In the remaining 7 articles the number of imputed variables could not be determined.

The majority of trials (55/73, 75%) imputed incomplete outcome variables, while only a few observational studies (9/30, 30%) imputed incomplete outcomes. In contrast, imputing missing data in covariates was more common in observational studies (n = 21, 70%), but less common in trials (n = 13, 18%). Just over half of the studies that imputed covariates imputed only one or two covariates (18 out of 34).

### Imputation details

The details of the imputation process extracted from the identified articles are summarised in Table 4. Of the 103 articles, 40 (39%) provided no details of the imputation method, variables used in the imputation model or the number of imputations. Where the imputation method was clearly specified (38/103, 37%), 20 papers (53%) used MICE, 8 papers (21%) used MVNI, and one article (3%) used PMM. Two papers applied MICE as well as PMM, and the remaining 7 papers, used MI on the basis of propensity score or regression-based imputation. For the two main imputation methods, 12 out of 20 papers for MICE, and 5 out of 8 papers for MVNI imputed multiple variables with missing data.

Although the majority of papers did not explicitly state the imputation method used, this could be inferred in an additional 21 articles using the version of the imputation software (e.g. MI under chained equations was the only multivariable command available in Stata 10 and earlier versions), the default programme (e.g. IVEware, SAS callable software application implements MI using chained equations), or specific procedure (e.g. SAS Proc MI via MCMC algorithm assumes multivariate normal distribution for implementing MI).

Twelve of the articles (12%) transformed non-normal variables prior to imputation. The logarithm (*log*) transformation was the most commonly reported method for handling non-normally distributed variables (n = 8); one article used the *logit* transformation and the remaining 3 articles referred to normalising transformations (n = 2) or mentioned that a transformation was used if appropriate (n = 1).

Only 39 of the articles (38%) specified the variables included in the imputation model. Of these, in 10 articles the imputation model included auxiliary variables, in 4 the imputation model included interaction terms, and in 5 the imputation model included auxiliary variables and interactions. In the remaining 20 articles, no further information was provided on whether the variables included in the imputation model were auxiliary variables or interaction terms. Only a few trials (n = 5) stated that they imputed the missing data in each arm separately.

Less than half of the articles (n = 47/103, 46%) reported the number of imputations used. The number of imputations ranged from < = 5 (11 papers) to >100 (3 papers), with nearly half of the papers (n = 23) using 10 to 50 imputations.

Only 2 studies (2%) carried out any diagnostic checks of their imputation model, both of which compared the distribution of observed and imputed values. Differences between the results from a complete case analysis (or LOCF) and MI were presented in 62 articles (60%).

Seventy-six articles (74%) stated the software in which the MI was implemented; SAS (n = 33), Stata (n = 27), and R (n = 12) were the commonly most used packages.

### Analysis status of MI

In 38 studies (37%) MI was used as the primary analysis and in 66 studies (64%) MI was used as a secondary analysis. Where MI was implemented as a secondary analysis, complete case analysis was the most common method for handling missing data in the primary analysis (62 out of 66 articles).

Finally, of the 103 papers reviewed, only 3 papers (3%) performed a sensitivity analysis following MI to investigate the robustness of the MI estimate to departures from the MAR assumption. Of these, only one paper provided details about the sensitivity analysis approach. The other two papers gave no information on the method used in the sensitivity analysis.

## Discussion

Missing data frequently occur in clinical and epidemiological research. MI is now recognised as a flexible and efficient approach to deal with missing data and is widely employed by investigators in a wide variety of settings. In this review, we show that the application of MI has increased in research articles published in the *Lancet* and *NEJM* over the 5-year period from 2008 to 2013. This finding demonstrates the growing popularity of MI since the previous review by Mackinnon [10], that also observed a significant increase in use of MI between 2005 and 2008 in articles published in four major medical journals.

We identified 103 studies from these two high ranking journals that implemented MI in the statistical analyses. These papers represent approximately 3% of the total 3815 original research articles published between January 2008 and December 2013 (i.e. 58/1705 papers in the *Lancet* and 45/2110 papers in the *NEJM*) [29]. Since the majority of the identified papers were trials where the missing outcome variables were imputed, we re-emphasise the need for trialists to consider schemes to encourage maximum follow-up of participants at the pilot phase and design stage of the study [6]. This may minimize the amount and potential impact of missing data.

Despite the presence of guidelines and recommendations for clear documentation around the reporting of missing data [1-3,5,9,10,30,31], there remains poor reporting of the amount of missing data with only half of the articles in this review providing the proportion of the complete cases or the proportion of missing data for each variable of interest in the manuscript. This observation is consistent with the finding of Mackinnon [10], who found that just over half of the articles did not provide any information about the amount of missing data. When performing MI, one should clearly report the amount of missing data for each variable included in the analysis, or at least, state the number of cases with complete observations. Providing this information helps the reader to assess the validity of the results since a larger proportion of missing data may introduce a greater bias in the analysis.

Exploring differences between individuals with complete and incomplete data can be used to examine the validity of the MCAR assumption; however, few articles compared the characteristics of participants with complete and incomplete data. Moreover, less than one-third of the published papers had a statement about the assumption made regarding the missing data within the analysis. Collecting information on reasons for missing data is very valuable and can help determine the most plausible assumption underlying the missing data. Since the standard application of MI assumes that data are MAR, it is important for investigators to acknowledge this assumption when using MI and justify it. Given it is not possible to assess the validity of the MAR assumption, it is also important to assess the sensitivity of the results to this assumption by making alternative assumptions about the missing data, for example that data are missing not at random (MNAR), that is allowing the missingness to depend on the unobserved data.

Previously published reviews and guidelines of handling missing data suggest the importance of providing adequate information on the imputation process used in MI [5,10]. However, this was one of the greatest weaknesses we found in our review. While details of the imputation method were available in just over one-third of the articles, this information was often not explicitly stated and was instead derived from the details of the imputation software. It is important that the researcher performing MI recognise the impact of decisions made in setting up the imputation model on the MI results. Presenting details on how MI was carried out may provide insight regarding the distributional assumptions made for variables with missing data, so that the validity of the results can be assessed. Detailed information, required for all statistical analyses, also ensures reproducibility of the results.

In our review, approximately one-third of articles clearly specified the variables included in the imputation model, and only half of these commented on the inclusion of auxiliary variables or interaction terms. Obtaining information on auxiliary variables that are highly associated with the missingness mechanism and the incomplete variable, and incorporating them into the imputation model can make the MAR assumption more plausible. In addition, it can improve the accuracy of the imputations, and as a result, may reduce the bias and increase the precision of the estimates.

In line with the Mackinnon’s review [10], we found that less than half of the articles stated the number of imputations, with nearly half using 10 to 50 imputations. As mentioned earlier, applying an adequate number of imputations is recommended in order to avoid producing a large Monte Carlo error. Therefore, stating the number of imputations along with the percentage of missing data within each variable can help readers make a better judgment about the accuracy of the MI results.

Despite the recent acknowledgement about the importance of carrying out diagnostic checks of imputation models [135-138], only two articles carried out any form of diagnostic checking, both of which compared the distribution of the observed and imputed values.

The importance of reporting and explaining differences between the results of complete case analysis and MI was pointed out by Sterne et al. [5], and have been recently emphasised by Powney et al. [1] in a supporting guidance for handling missing data in longitudinal trials. However, our review shows that just under two-third of articles compared the estimates derived from a complete case analysis and MI.

While the findings of the paper published by Mackinnon [10] showed that MI was implemented as the primary analysis in just over half of the identified articles, we observed that MI was adopted as the secondary analysis in just under two-third of the studies, with complete case analysis generally being used as the primary analysis.

Disappointingly, the majority of the reviewed articles failed to recognise the importance of conducting sensitivity analyses regarding the MAR assumption following MI. More specifically, only three articles explored the sensitivity of the results to departures from MAR, of which only one article [128] provided details of the estimates derived. We understand that there is a lack of explicit guidelines in the literature for conducting sensitivity analyses within the MI framework. The development of this area is a subject of ongoing research. We believe that performing sensitivity analyses regarding the MAR assumption post-MI should be regarded as an essential part of the analysis to assess the robustness of the conclusions to plausible departures from this critical assumption.

It is important to acknowledge that many journals have strict word limits for original research articles, including a maximum number of tables and figures that can be included. For example, in the *Lancet* the word limit is 3000 words, and in the *NEJM* it is 2700 words for Original and Special articles. This makes it difficult to include all of the details surrounding the imputation procedure. However, this information can be reported in a previously published “baseline” or “protocol” paper, or included as online supplements, especially in such journals with very tight word limits. In his recent paper, Ware [7] suggested some improvements in journal policies regarding the reporting of clinical trials and observational studies in presence of missing data, in addition to the documentation of details about the methods for handling missing data. As noted by Ware [7], journal authors will be required to state clearly the assumptions made about the missing data and justify them, and may be requested to conduct sensitivity analyses where appropriate. In addition, authors will be expected to provide supplementary appendices in which the details of the method for analysis of missing data are clearly explained. It is important to note that while just over two-thirds of the identified articles in this review included supplementary materials or web appendices, very few articles provided a complete description of the missing data and the MI procedure according to the available reporting guidelines.

## Conclusions

This review of articles published (in the *Lancet* and *NEJM*) in the past 5 years (2008–2013) has highlighted the continued inadequacy of reporting information about missing data and the MI procedure in research articles that use MI. Although our review showed improvements in reporting since the Mackinnon review of articles published between 1994 (earliest MI publication) and 2008, many articles are still not following the guidelines and recommendations for the reporting of missing data and statistical analyses using MI.

It is essential that researchers are aware of the issues that may arise when implementing MI, and more importantly that biased and imprecise results may be obtained if the imputation model is misspecified. Authors are encouraged to provide information on missing data, include details of the analyses when using MI, explore the impact of missing data on their results, and assess the sensitivity of the MI results to plausible departures from the MAR assumption. Providing detailed information on all of the above allows readers to make an informed decision about the quality of the study results, appropriateness of the imputation process, and validity of the results obtained from MI.

## Additional files

Additional file 1: Table S1. PRISMA checklist for reporting systematic reviews. Additional file 2: Table S2. Details of the articles included in the systematic review and the corresponding reference list [32-134].

## Abbreviations

CC: Complete case
LOCF: Last observation carried forward
MAR: Missing at random
MCAR: Missing completely at random
MCMC: Markov chain Monte Carlo
MI: Multiple imputation
MICE: Multivariate imputation by chained equations
MNAR: Missing not at random
MVNI: Multivariate normal imputation
NEJM: New England Journal of Medicine
PMM: Predictive mean matching
RCT: Randomised controlled trial
STROBE: Strengthening the Reporting of Observational Studies in Epidemiology
