# Supplementary material for: The rise of multiple imputation: a review of the reporting and implementation of the method in medical research
Source: BMC Med Res Methodol. 2015 Apr 7;15:30. doi: 10.1186/s12874-015-0022-1 (PMC4396150; doi:10.1186/s12874-015-0022-1)
Supplement: Additional file 2: Table S2. — Details of the articles included in the systematic review and the corresponding reference list [32-134]. [file 12874_2015_22_MOESM2_ESM.docx]

Table S2: Detailed characteristics of the articles included in the systematic review.

| **Author**  **(Year)** | **Study type** | **Percentage of**  **complete cases** | **Number of variables imputed** | **Imputation method** | **Number of imputation** | **Imputation software** | **MI used for secondary analysis** |
| --- | --- | --- | --- | --- | --- | --- | --- |
| Abdulla  (2008) [32] | Trial | NA | 1 | NA | NA | NA | Yes |
| Acker  (2013) [33] | Trial | NA | 1 | NA | NA | NA | No |
| Alio  (2009) [34] | Observational | NA | 2 | NA | NA | SAS | Yes |
| Ang  (2010) [35] | Observational | NA | 2 | MVNI^a^ | 20 | SAS | Yes |
| Armstrong  (2013) [36] | Trial | NA | NA | NA | 100 | R | Yes |
| Beasley  (2008) [37] | Observational | 54 | Cannot be verified | NA | NA | NA | Yes |
| Bergenstal  (2010) [38] | Trial | 90 | 1 | Regression_based MI | NA | SAS | Yes |
| Bergenstal  (2013) [39] | Trial | NA | 1 | NA | 5 | SAS | No |
| Boonen  (2012) [40] | Trial | NA | 1 | Regression_based MI | 200 | NA | Yes |
| Brott  (2010) [41] | Trial | NA | NA | NA | NA | NA | Yes |
| Buchbinder  (2008) [42] | Trial | NA | 1 | NA | NA | NA | No |
| Buse  (2009) [43] | Trial | NA | NA | NA | NA | NA | Yes |
| Carlsson  (2012) [44] | Trial | NA | 2 | MICE | 30 | Stata | Yes |
| Caroli  (2013) [45] | Trial | 89 | NA | MICE | NA | Stata | No |
| **Author**  **(Year)** | **Study type** | **Percentage of complete cases** | **Number of variables imputed** | **Imputation method** | **Number of imputation** | **Imputation software** | **MI used for secondary analysis** |
| Chan  (2013) [46] | Observational | NA | 2 | NA | NA | SAS^b^ | Yes |
| Chew  (2010) [47] | Trial | 82 | 1 | Regression_based MI | NA | SAS | Yes |
| Corbacioglu  (2012) [48] | Trial | 95 | 1 | NA | NA | SAS | Yes |
| Day  (2013) [49] | Trial | NA | >2 | MICE | NA | R | No |
| de Ruyter  (2012) [50] | Trial | 74 | 1 | MICE | 30 | R | Yes |
| el Barzouhi  (2013) [51] | Trial | NA | >2 | Model-based MI | NA | NA | No |
| Farooq  (2013) [52] | Observational | NA | >2 | MICE | NA | R | No |
| Feldman  (2011) [53] | Trial | 97 | 2 | NA | NA | SAS | Yes |
| Free  (2011) [54] | Trial | 95 | >2 | MICE | 100 | Stata | No |
| Funk  (2010) [55] | Observational | 28 | 1 | NA | 100 | SAS | Yes |
| Gadde  (2011) [56] | Trial | 61 | >2 | MVNI^a^ | 5 | NA | Yes |
| Gebre  (2012) [57] | Trial | 98 | 1 | MICE | NA | R | No |
| Gill  (2010) [58] | Observational | NA | 1 | MVNI | 100 | SAS | No |
| Girotra  (2012) [59] | Observational | NA | >2 | MICE | NA | SAS^b^ | Yes |
| Gler  (2012) [60] | Trial | NA | 2 | NA | NA | NA | Yes |
| **Author**  **(Year)** | **Study type** | **Percentage of complete cases** | **Number of variables imputed** | **Imputation method** | **Number of imputation** | **Imputation software** | **MI used for secondary analysis** |
| Green  (2010) [61] | Trial | NA | >2 | MICE-PMM | 500 | SAS | No |
| Green  (2009) [62] | Trial | NA | NA | MVNI^a^ | NA | SAS | No |
| Gupta  (2013) [63] | Trial | NA | 1 | NA | 20 | Stata | Yes |
| Gutiérrez  (2008) [64] | Observational | NA | NA | NA | NA | Stata | Yes |
| Hanney  (2012) [65] | Trial | 84 | 1 | NA | NA | Stata | Yes |
| Hegarty  (2013) [66] | Trial | NA | >2 | MICE | 50 | Stata/SPSS | Yes |
| Hill  (2011) [67] | Trial | 76 | Cannot be verified | NA | 5 | NA | No |
| Holman  (2009) [68] | Trial | NA | NA | MVNI^a^ | 5 | Stata | No |
| Hunt  (2013) [69] | Observational | NA | 2 | MICE | 100 | SAS | Yes |
| Jabre  (2013) [70] | Trial | 83 | 1 | NA | NA | SAS | No |
| Jacobs  (2013) [71] | Trial | 87 | 1 | Propensity score | 10 | Stata | No |
| Katz  (2013) [72] | Observational | NA | 1 | MI command | NA | Stata | No |
| Kelly  (2012) [73] | Trial | 91 | >2 | MVNI | 13 | Stata | Yes |
| Kessler  (2009) [74] | Trial | NA | >2 | MICE | 25 | R | Yes |
| King  (2009) [75] | Trial | 92 | >2 | MVNI | 5 | SAS | No |
| **Author**  **(Year)** | **Study type** | **Percentage of complete cases** | **Number of variables imputed** | **Imputation method** | **Number of imputation** | **Imputation software** | **MI used for secondary analysis** |
| Kinmonth  (2008) [76] | Trial | 88 | 1 | NA | NA | S-plus | Yes |
| Kirby  (2009) [77] | Trial | NA | 2 | NA | NA | Stata/SPSS | No |
| Labrie  (2013) [78] | Trial | 80 | NA | PMM | 10 | SPSS | Yes |
| Lamb  (2009) [79] | Trial | NA | >2 | MICE | NA | Stata | Yes |
| Lamb  (2013) [80] | Trial | 68 | >2 | NA | NA | Stata | No |
| Lamb  (2010) [81] | Trial | 85 | NA | NA | NA | SAS/Stata | Yes |
| Lawlor  (2013) [82] | Observational | 72 | >2 | MICE | 50 | Stata | Yes |
| Lester  (2010) [83] | Trial | NA | >2 | Proc MI | 5 | SAS | Yes |
| Lidegaard  (2012) [84] | Observational | 70 | 1 | Regression_based MI | 100 | NA | Yes |
| Liem  (2013) [85] | Trial | NA | 1 | NA | NA | R | Yes |
| Lim  (2008) [86] | Observational | NA | 1 | NA | 100 | R^c^ | Yes |
| Lo  (2010) [87] | Trial | NA | NA | NA | NA | SAS | Yes |
| Lorenz  (2012) [88] | Observational | NA | Cannot be verified | NA | 10 | Stata/SPSS | No |
| Lu  (2010) [89] | Observational | 39 | 2 | NA | 100 | R^c^ | No |
| Marcus  (2013) [90] | Trial | 88 | 1 | MVNI^a^ | 10 | SAS | Yes |
| **Author**  **(Year)** | **Study type** | **Percentage of complete cases** | **Number of variables imputed** | **Imputation method** | **Number of imputation** | **Imputation software** | **MI used for secondary analysis** |
| Martin  (2011) [91] | Trial | NA | Cannot be verified | Propensity score/regression modelling | NA | SAS | Yes |
| Mauer  (2009) [92] | Trial | NA | 2 | NA | NA | SAS | Yes |
| May  (2010) [93] | Observational | 39 | >2 | MICE-PMM | 25 | Stata | No |
| McManus  (2010) [94] | Trial | 91 | 1 | NA | NA | Minitab/SPSS | Yes |
| Melhuish  (2008) [95] | Trial | NA | >2 | NA | 10 | SAS^b^ | Yes |
| Milstone  (2013) [96] | Trial | NA | 1 | NA | NA | R/Stata | Yes |
| Montalescot  (2011) [97] | Trial | 97 | 2 | Proc MI | NA | SAS | Yes |
| Mozaffarian  (2011) [98] | Observational | NA | Cannot be verified | NA | NA | SAS | No |
| Odd  (2009) [99] | Observational | 42 | >2 | MICE | NA | Stata | Yes |
| Olanow  (2009) [100] | Trial | 84 | Cannot be verified | NA | NA | NA | Yes |
| Pandharipande  (2013) [101] | Observational | NA | >2 | NA | NA | R | No |
| Parker  (2010) [102] | Trial | NA | 1 | MICE | 10 | Stata | No |
| Patel  (2010) [103] | Trial | 87 | 1 | MICE | NA | Stata | Yes |
| Peek  (2009) [104] | Trial | NA | >2 | NA | NA | SOLAS | Yes |
| Pimentel  (2011) [105] | Trial | NA | 2 | NA | NA | NA | Yes |
| **Author**  **(Year)** | **Study type** | **Percentage of complete cases** | **Number of variables imputed** | **Imputation method** | **Number of imputation** | **Imputation software** | **MI used for secondary analysis** |
| Price  (2011) [106] | Trial | 98 | 2 | MVNI | 10 | SAS | No |
| Pronyk  (2012) [107] | Trial | NA | Cannot be verified | NA | NA | SAS | No |
| Puhan  (2009) [108] | Observational | NA | 2 | NA | 50 | Stata | No |
| Roberts  (2012) [109] | Trial | NA | NA | Proc MI | NA | SAS | No |
| Sitas  (2013) [110] | Observational | 28 | 2 | MICE | 10 | Stata | Yes |
| Smith  (2011) [111] | Trial | NA | >2 | Proc MI | 100 | SAS | Yes |
| Solomon  (2012) [112] | Trial | NA | 1 | NA | NA | Stata | Yes |
| Sommers  (2012) [113] | Trial | NA | 1 | Regression_based MI | NA | NA | Yes |
| Stanworth  (2013) [114] | Trial | 99 | 1 | MICE | 50 | Stata | No |
| Sterne  (2009) [115] | Observational | NA | >2 | NA | 25 | SAS/Stata | Yes |
| Strang  (2010) [116] | Trial | 47 | 1 | MICE | 100 | Stata | No |
| Subak  (2009) [117] | Trial | NA | >2 | NA | NA | NA | No |
| Tanser  (2011) [118] | Observational | 74 | >2 | NA | 5 | Stata | Yes |
| Thabut  (2008) [119] | Observational | NA | NA | MICE | 5 | R | No |
| Underwood  (2013) [120] | Trial | NA | 2 | NA | 5 | Stata | Yes |
| **Author**  **(Year)** | **Study type** | **Percentage of complete cases** | **Number of variables imputed** | **Imputation method** | **Number of imputation** | **Imputation software** | **MI used for secondary analysis** |
| Vaucher  (2012) [121] | Trial | 94 | 1 | NA | NA | NA | Yes |
| Visco  (2012) [122] | Trial | 91 | 1 | NA | NA | SAS | Yes |
| Wadden  (2011) [123] | Trial | 86 | 1 | NA | NA | NA | Yes |
| Walkup  (2008) [124] | Trial | NA | NA | NA | NA | SAS | Yes |
| Walz  (2010) [125] | Trial | 80 | 1 | NA | NA | NA | No |
| Wang  (2011) [126] | Trial | NA | NA | NA | NA | SAS | Yes |
| Weeks  (2012) [127] | Observational | NA | >2 | NA | NA | SAS/Stata | Yes |
| Weintraub  (2008) [128] | Trial | NA | >2 | MICE | 5 | Stata | Yes |
| Weiser  (2008) [129] | Observational | 29 | 1 | NA | 300 | SAS | Yes |
| Westerlund  (2009) [130] | Observational | NA | 1 | Proc MI | 5 | SAS | Yes |
| Wherrett  (2011) [131] | Trial | NA | 1 | NA | NA | S-plus | No |
| Wiles  (2013) [132] | Trial | NA | 1 | MICE | 25 | Stata | Yes |
| Yu  (2013) [133] | Observational | NA | >2 | NA | 20 | R | Yes |
| Zeig-OweNA  (2011) [134] | Observational | NA | 1 | NA | 10 | SAS | No |

Abbreviations: NA- not available, MI- multiple imputation, MICE- multiple imputation by chained equations, MVNI- multivariate normal imputation, PMM- predictive mean matching, MCMC-Markov chain Monte Carlo algorithm, CC- complete case, LOCF- last observation carried forward.

^a^ MI was used via MCMC algorithm.

^b^IVEware package was used.

^c^ Amelia package was used.

**References**

32. Abdulla S, Sagara I, Borrmann S, D'Alessandro U, González R, Hamel M, Ogutu B, Mårtensson A, Lyimo J, Maiga H, Sasi P, Nahum A, Bassat Q, Juma E, Otieno L, Björkman A, Beck HP, Andriano K, Cousin M, Lefèvre G, Ubben D, Premji Z: **Efficacy and safety of artemether-lumefantrine dispersible tablets compared with crushed commercial tablets in African infants and children with uncomplicated malaria: a randomised, single-blind, multicentre trial.** The Lancet 2008, **372**(9652):1819-1827.

33. Acker MA, Parides MK, Perrault LP, Moskowitz AJ, Gelijns AC, Voisine P, Smith PK, Hung JW, Blackstone EH, Puskas JD, Argenziano M, Gammie JS, Mack M, Ascheim DD, Bagiella E, Moquete EG, Ferguson TB, Horvath KA, Geller NL, Miller MA, Woo YJ, D'Alessandro DA, Ailawadi G, Dagenais F, Gardner TJ, O'Gara PT, Michler RE, Kron IL: **Mitral-valve repair versus replacement for severe ischemic mitral regurgitation.** N Engl J Med 2014, **370**(1):23 (Epub 2013).

34. Alio AP, Nana PN, Salihu HM: **Spousal violence and potentially preventable single and recurrent spontaneous fetal loss in an African setting: cross-sectional study.** The Lancet 2009, **373**(9660):318-324.

35. Ang KK, Harris J, Wheeler R, Weber R, Rosenthal DI, Nguyen-Tân PF, Westra WH, Chung CH, Jordan RC, Lu C, Kim H, Axelrod R, Silverman CC, Redmond KP, Gillison ML: **Human papillomavirus and survival of patients with oropharyngeal cancer.** N Engl J Med 2010, **363**(1):24-35.

36. Armstrong PW, Gershlick AH, Goldstein P, Wilcox R, Danays T, Lambert Y, Sulimov V, Rosell Ortiz F, Ostojic M, Welsh RC, Carvalho AC, Nanas J, Arntz H, Halvorsen S, Huber K, Grajek S, Fresco C, Bluhmki E, Regelin A, Vandenberghe K, Bogaerts K, Van dW: **Fibrinolysis or Primary PCI in ST-Segment Elevation Myocardial Infarction.** N Engl J Med 2013, **368**(15):1379-1387.

37. Beasley R, Clayton T, Crane J, von Mutius E, Lai CK, Montefort S, Stewart A: **Association between paracetamol use in infancy and childhood, and risk of asthma, rhinoconjunctivitis, and eczema in children aged 6–7 years: analysis from Phase Three of the ISAAC programme.** The Lancet 2008, **372**(9643):1039-1048.

38. Bergenstal RM, Tamborlane WV, Ahmann A, Buse JB, Dailey G, Davis SN, Joyce C, Peoples T, Perkins BA, Welsh JB, Willi SM, Wood MA: **Effectiveness of Sensor-Augmented Insulin-Pump Therapy in Type 1 Diabetes.** N Engl J Med 2010, **363**(4):311-320.

39. Bergenstal RM, Klonoff DC, Garg SK, Bode BW, Meredith M, Slover RH, Ahmann AJ, Welsh JB, Lee SW, Kaufman FR: **Threshold-Based Insulin-Pump Interruption for Reduction of Hypoglycemia.** N Engl J Med 2013, **369**(3):224-232.

40. Boonen S, Reginster J, Kaufman J, Lippuner K, Zanchetta J, Langdahl B, Rizzoli R, Lipschitz S, Dimai HP, Witvrouw R, Eriksen E, Brixen K, Russo L, Claessens F, Papanastasiou P, Antunez O, Su G, Bucci-Rechtweg C, Hruska J, Incera E, Vanderschueren D, Orwoll E: **Fracture Risk and Zoledronic Acid Therapy in Men with Osteoporosis.** N Engl J Med 2012, **367**(18):1714-1723.

41. Brott TG, Hobson II RW, Howard G, Roubin GS, Clark WM, Brooks W, Mackey A, Hill MD, Leimgruber PP, Sheffet AJ, Howard VJ, Moore WS, Voeks JH, Hopkins LN, Cutlip DE, Cohen DJ, Popma JJ, Ferguson RD, Cohen SN, Blackshear JL, Silver FL, Mohr JP, Lal BK, Meschia JF: **Stenting versus endarterectomy for treatment of carotid-artery stenosis.** N Engl J Med 2010, **363**(1):11-23.

42. Buchbinder SP, Mehrotra DV, Duerr A, Fitzgerald DW, Mogg R, Li D, Gilbert PB, Lama JR, Marmor M, del Rio C, McElrath MJ, Casimiro DR, Gottesdiener KM, Chodakewitz JA, Corey L, Robertson MN: **Efficacy assessment of a cell-mediated immunity HIV-1 vaccine (the Step Study): a double-blind, randomised, placebo-controlled, test-of-concept trial.** The Lancet **372**(9653):1881-1893.

43. Buse JB, Rosenstock J, Sesti G, Schmidt WE, Montanya E, Brett JH, Zychma M, Blonde L: **Liraglutide once a day versus exenatide twice a day for type 2 diabetes: a 26-week randomised, parallel-group, multinational, open-label trial (LEAD-6).** The Lancet 2009, **374**(9683):39-47.

44. Carlsson LMS, Peltonen M, Ahlin S, Anveden Å, Bouchard C, Carlsson B, Jacobson P, Lönroth H, Maglio C, Näslund I, Pirazzi C, Romeo S, Sjöholm K, Sjöström E, Wedel H, Svensson P, Sjöström L: **Bariatric Surgery and Prevention of Type 2 Diabetes in Swedish Obese Subjects.** N Engl J Med 2012, **367**(8):695-704.

45. Caroli A, Perico N, Perna A, Antiga L, Brambilla P, Pisani A, Visciano B, Imbriaco M, Messa P, Cerutti R, Dugo M, Cancian L, Buongiorno E, De Pascalis A, Gaspari F, Carrara F, Rubis N, Prandini S, Remuzzi A, Remuzzi G, Ruggenenti P: **Effect of longacting somatostatin analogue on kidney and cyst growth in autosomal dominant polycystic kidney disease (ALADIN): a randomised, placebo-controlled, multicentre trial.** The Lancet 2013, **382**(9903):1485-1495.

46. Chan PS, Nallamothu BK, Krumholz HM, Spertus JA, Li Y, Hammill BG, Curtis LH: **Long-Term Outcomes in Elderly Survivors of In-Hospital Cardiac Arrest.** N Engl J Med 2013, **368**(11):1019-1026.

47. Chew EY, Ambrosius WT, Davis MD, Danis RP, Gangaputra S, Greven CM, Hubbard L, Esser BA, Lovato JF, Perdue LH, Goff Jr. DC, Cushman WC, Ginsberg HN, Elam MB, Genuth S, Gerstein HC, Schubart U, Fine LJ: **Effects of medical therapies on retinopathy progression in type 2 diabetes.** N Engl J Med 2010, **363**(3):233-244.

48. Corbacioglu S, Cesaro S, Faraci M, Valteau-Couanet D, Gruhn B, Rovelli A, Boelens JJ, Hewitt A, Schrum J, Schulz AS, Müller I, Stein J, Wynn R, Greil J, Sykora K, Matthes-Martin S, Führer M, O'Meara A, Toporski J, Sedlacek P, Schlegel PG, Ehlert K, Fasth A, Winiarski J, Arvidson J, Mauz-Körholz C, Ozsahin H, Schrauder A, Bader P, Massaro J, D'Agostino R, Hoyle M, Iacobelli M, Debatin K, Peters C, Dini G: **Defibrotide for prophylaxis of hepatic veno-occlusive disease in paediatric haemopoietic stem-cell transplantation: an open-label, phase 3, randomised controlled trial.** The Lancet 2012, **379**(9823):1301-1309.

49. Day JN, Chau TTH, Wolbers M, Mai PP, Dung NT, Mai NH, Phu NH, Nghia HD, Phong ND, Thai CQ, Thai LH, Chuong LV, Sinh DX, Duong VA, Hoang TN, Diep PT, Campbell JI, Sieu TPM, Baker SG, Chau NVV, Hien TT, Lalloo DG, Farrar JJ: **Combination Antifungal Therapy for Cryptococcal Meningitis.** N Engl J Med 2013, **368**(14):1291-1302.

50. de Ruyter JC, Olthof MR, Seidell JC, Katan MB: **A Trial of Sugar-free or Sugar-Sweetened Beverages and Body Weight in Children.** N Engl J Med 2012, **367**(15):1397-1406.

51. el Barzouhi A, Vleggeert-Lankamp C, Lycklama à Nijeholt, Geert J., Van dK, van dH, Jacobs WCH, Koes BW, Peul WC: **Magnetic Resonance Imaging in Follow-up Assessment of Sciatica.** N Engl J Med 2013, **368**(11):999-1007.

52. Farooq V, van Klaveren D, Steyerberg EW, Meliga E, Vergouwe Y, Chieffo A, Kappetein AP, Colombo A, Holmes Jr DR, Mack M, Feldman T, Morice M, Ståhle E, Onuma Y, Morel M, Garcia-Garcia HM, van Es GA, Dawkins KD, Mohr FW, Serruys PW: **Anatomical and clinical characteristics to guide decision making between coronary artery bypass surgery and percutaneous coronary intervention for individual patients: development and validation of SYNTAX score II.** The Lancet **381**(9867):639-650.

53. Feldman T, Foster E, Glower DD, Kar S, Rinaldi MJ, Fail PS, Smalling RW, Siegel R, Rose GA, Engeron E, Loghin C, Trento A, Skipper ER, Fudge T, Letsou GV, Massaro JM, Mauri L: **Percutaneous Repair or Surgery for Mitral Regurgitation.** N Engl J Med 2011, **364**(15):1395-1406.

54. Free C, Knight R, Robertson S, Whittaker R, Edwards P, Zhou W, Rodgers A, Cairns J, Kenward MG, Roberts I: **Smoking cessation support delivered via mobile phone text messaging (txt2stop): A single-blind, randomised trial.** The Lancet 2011, **378**(9785):49-55.

55. Funk LM, Weiser TG, Berry WR, Lipsitz SR, Merry AF, Enright AC, Wilson IH, Dziekan G, Gawande AA: **Global operating theatre distribution and pulse oximetry supply: An estimation from reported data.** The Lancet 2010, **376**(9746):1055-1061.

56. Gadde KM, Allison DB, Ryan DH, Peterson CA, Troupin B, Schwiers ML, Day WW: **Effects of low-dose, controlled-release, phentermine plus topiramate combination on weight and associated comorbidities in overweight and obese adults (CONQUER): a randomised, placebo-controlled, phase 3 trial.** The Lancet 2011, **377**(9774):1341-1352.

57. Gebre T, Ayele B, Zerihun M, Genet A, Stoller NE, Zhou Z, House JI, Yu SN, Ray KJ, Emerson PM, Keenan JD, Porco TC, Lietman TM, Gaynor BD: **Comparison of annual versus twice-yearly mass azithromycin treatment for hyperendemic trachoma in Ethiopia: a cluster-randomised trial.** The Lancet 2012, **379**(9811):143-151.

58. Gill TM, Gahbauer EA, Han L, Allore HG: **Trajectories of Disability in the Last Year of Life.** N Engl J Med 2010, **362**(13):1173-1180.

59. Girotra S, Nallamothu BK, Spertus JA, Li Y, Krumholz HM, Chan PS: **Trends in Survival after In-Hospital Cardiac Arrest.** N Engl J Med 2012, **367**(20):1912-1920.

60. Gler MT, Skripconoka V, Sanchez-Garavito E, Xiao H, Cabrera-Rivero J, Vargas-Vasquez D, Gao M, Awad M, Park S, Shim TS, Suh GY, Danilovits M, Ogata H, Kurve A, Chang J, Suzuki K, Tupasi T, Koh W, Seaworth B, Geiter LJ, Wells CD: **Delamanid for Multidrug-Resistant Pulmonary Tuberculosis.** N Engl J Med 2012, **366**(23):2151-2160.

61. Green J, Charman T, McConachie H, Aldred C, Slonims V, Howlin P, Le Couteur A, Leadbitter K, Hudry K, Byford S, Barrett B, Temple K, Macdonald W, Pickles A: **Parent-mediated communication-focused treatment in children with autism (PACT): a randomised controlled trial.** The Lancet 2010, **375**(9732):2152-2160.

62. Green RC, Roberts JS, Cupples LA, Relkin NR, Whitehouse PJ, Brown T, Eckert SL, Butson M, Sadovnick AD, Quaid KA, Chen C, Cook-Deegan R, Farrer LA: **Disclosure of APOE Genotype for Risk of Alzheimer's Disease.** N Engl J Med 2009, **361**(3):245-254.

63. Gupta J, Kai J, Middleton L, Pattison H, Gray R, Daniels J: **Levonorgestrel intrauterine system versus medical therapy for menorrhagia.** N Engl J Med 2013, **368**(2):128-137.

64. Gutiérrez OM, Mannstadt M, Isakova T, Rauh-Hain J, Tamez H, Shah A, Smith K, Lee H, Thadhani R, Jüppner H, Wolf M: **Fibroblast Growth Factor 23 and Mortality among Patients Undergoing Hemodialysis.** N Engl J Med 2008, **359**(6):584-592.

65. Hanney M, Prasher V, Williams N, Jones EL, Aarsland D, Corbett A, Lawrence D, Yu L, Tyrer S, Francis PT, Johnson T, Bullock R, Ballard C: **Memantine for dementia in adults older than 40 years with Down's syndrome (MEADOWS): a randomised, double-blind, placebo-controlled trial.** The Lancet 2012, **379**(9815):528-536.

66. Hegarty K, O'Doherty L, Taft A, Chondros P, Brown S, Valpied J, Astbury J, Taker A, Gold L, Feder G, Gunn J: **Screening and counselling in the primary care setting for women who have experienced intimate partner violence (WEAVE): a cluster randomised controlled trial.** Lancet 2013, **382**(9888):249-258.

67. Hill JC, Whitehurst DG, Lewis M, Bryan S, Dunn KM, Foster NE, Konstantinou K, Main CJ, Mason E, Somerville S, Sowden G, Vohora K, Hay EM: **Comparison of stratified primary care management for low back pain with current best practice (STarT Back): a randomised controlled trial.** Lancet 2011, **378**(9802):1560-1571.

68. Holman RR, Farmer AJ, Davies MJ, Levy JC, Darbyshire JL, Keenan JF, Paul SK: **Three-year efficacy of complex insulin regimens in type 2 diabetes.** N Engl J Med 2009, **361**(18):1736-1747.

69. Hunt LP, Ben-Shlomo Y, Clark EM, Dieppe P, Judge A, MacGregor AJ, Tobias JH, Vernon K, Blom AW: **90-day mortality after 409 096 total hip replacements for osteoarthritis, from the National Joint Registry for England and Wales: a retrospective analysis.** The Lancet **382**(9898):1097-1104.

70. Jabre P, Belpomme V, Azoulay E, Jacob L, Bertrand L, Lapostolle F, Tazarourte K, Bouilleau G, Pinaud V, Broche C, Normand D, Baubet T, Ricard-Hibon A, Istria J, Beltramini A, Alheritiere A, Assez N, Nace L, Vivien B, Turi L, Launay S, Desmaizieres M, Borron SW, Vicaut E, Adnet F: **Family Presence during Cardiopulmonary Resuscitation.** N Engl J Med 2013, **368**(11):1008-1018.

71. Jacobs AK, Normand ST, Massaro JM, Cutlip DE, Carrozza JP, Marks AD, Murphy N, Romm IK, Biondolillo M, Mauri L: **Nonemergency PCI at Hospitals with or without On-Site Cardiac Surgery.** N Engl J Med 2013, **368**(16):1498-1508.

72. Katz J, Lee AC, Kozuki N, Lawn JE, Cousens S, Blencowe H, Ezzati M, Bhutta ZA, Marchant T, Willey BA, Adair L, Barros F, Baqui AH, Christian P, Fawzi W, Gonzalez R, Humphrey J, Huybregts L, Kolsteren P, Mongkolchati A, Mullany LC, Ndyomugyenyi R, Nien JK, Osrin D, Roberfroid D, Sania A, Schmiegelow C, Silveira MF, Tielsch J, Vaidya A, Velaphi SC, Victora CG, Watson-Jones D, Black RE: **Mortality risk in preterm and small-for-gestational-age infants in low-income and middle-income countries: a pooled country analysis.** The Lancet 2013, **382**(9890):417-425.

73. Kelly HW, Sternberg AL, Lescher R, Fuhlbrigge AL, Williams P, Zeiger RS, Raissy HH, Van Natta ML, Tonascia J, Strunk RC: **Effect of inhaled glucocorticoids in childhood on adult height.** N Engl J Med 2012, **367**(10):904-912.

74. Kessler D, Lewis G, Kaur S, Wiles N, King M, Weich S, Sharp DJ, Araya R, Hollinghurst S, Peters TJ: **Therapist-delivered internet psychotherapy for depression in primary care: a randomised controlled trial.** The Lancet 2009, **374**(9690):628-634.

75. King G, Gakidou E, Imai K, Lakin J, Moore RT, Nall C, Ravishankar N, Vargas M, Téllez-Rojo MM, Ávila JEH, Ávila MH, Llamas HH: **Public policy for the poor? A randomised assessment of the Mexican universal health insurance programme.** The Lancet 2009, **373**(9673):1447-1454.

76. Kinmonth A, Wareham NJ, Hardeman W, Sutton S, Prevost AT, Fanshawe T, Williams KM, Ekelund U, Spiegelhalter D, Griffin SJ: **Efficacy of a theory-based behavioural intervention to increase physical activity in an at-risk group in primary care (ProActive UK): a randomised trial.** The Lancet 2008, **371**(9606):41-48.

77. Kirby MJ, Ameh D, Bottomley C, Green C, Jawara M, Milligan PJ, Snell PC, Conway DJ, Lindsay SW: **Effect of two different house screening interventions on exposure to malaria vectors and on anaemia in children in The Gambia: a randomised controlled trial.** The Lancet 2009, **374**(9694):998-1009.

78. Labrie J, Berghmans BLCM, Fischer K, Milani AL, van dW, Smalbraak DJC, Vollebregt A, Schellart RP, Graziosi GCM, van dP, Brouns JFGM, Tiersma ES, Groenendijk AG, Scholten P, Mol BW, Blokhuis EE, Adriaanse AH, Schram A, Roovers JWR, Lagro-Janssen A, van dV: **Surgery versus Physiotherapy for Stress Urinary Incontinence.** N Engl J Med 2013, **369**(12):1124-1133.

79. Lamb SE, Marsh JL, Hutton JL, Nakash R, Cooke MW: **Mechanical supports for acute, severe ankle sprain: a pragmatic, multicentre, randomised controlled trial.** The Lancet 2009, **373**(9663):575-581.

80. Lamb SE, Gates S, Williams MA, Williamson EM, Mt-Isa S, Withers EJ, Castelnuovo E, Smith J, Ashby D, Cooke MW, Petrou S, Underwood MR: **Emergency department treatments and physiotherapy for acute whiplash: A pragmatic, two-step, randomised controlled trial.** The Lancet 2013, **381**(9866):546-556.

81. Lamb SE, Hansen Z, Lall R, Castelnuovo E, Withers EJ, Nichols V, Potter R, Underwood MR: **Group cognitive behavioural treatment for low-back pain in primary care: a randomised controlled trial and cost-effectiveness analysis.** The Lancet 2010, **375**(9718):916-923.

82. Lawlor DA, Wills AK, Fraser A, Sayers A, Fraser WD, Tobias JH: **Association of maternal vitamin D status during pregnancy with bone-mineral content in offspring: a prospective cohort study.** Lancet 2013, **381**(9884):2176-2183.

83. Lester RT, Ritvo P, Mills EJ, Kariri A, Karanja S, Chung MH, Jack W, Habyarimana J, Sadatsafavi M, Najafzadeh M, Marra CA, Estambale B, Ngugi E, Ball TB, Thabane L, Gelmon LJ, Kimani J, Ackers M, Plummer FA: **Effects of a mobile phone short message service on antiretroviral treatment adherence in Kenya (WelTel Kenya1): a randomised trial.** The Lancet **376**(9755):1838-1845.

84. Lidegaard Ø, Løkkegaard E, Jensen A, Skovlund CW, Keiding N: **Thrombotic stroke and myocardial infarction with hormonal contraception.** N Engl J Med 2012, **366**(24):2257-2266.

85. Liem S, Schuit E, Hegeman M, Bais J, de Boer K, Bloemenkamp K, Brons J, Duvekot H, Bijvank BN, Franssen M, Gaugler I, de Graaf I, Oudijk M, Papatsonis D, Pernet P, Porath M, Scheepers L, Sikkema M, Sporken J, Visser H, van Wijngaarden W, Woiski M, van Pampus M, Mol BW, Bekedam D: **Cervical pessaries for prevention of preterm birth in women with a multiple pregnancy (ProTWIN): a multicentre, open-label randomised controlled trial.** The Lancet 2013, **382**(9901):1341-1349.

86. Lim SS, Stein DB, Charrow A, Murray CJ: **Tracking progress towards universal childhood immunisation and the impact of global initiatives: a systematic analysis of three-dose diphtheria, tetanus, and pertussis immunisation coverage.** The Lancet 2008, **372**(9655):2031-2046.

87. Lo AC, Guarino PD, Richards LG, Haselkorn JK, Wittenberg GF, Federman DG, Ringer RJ, Wagner TH, Krebs HI, Volpe BT, Bever CT, Bravata DM, Duncan PW, Corn BH, Maffucci AD, Nadeau SE, Conroy SS, Powell JM, Huang GD, Peduzzi P: **Robot-Assisted Therapy for Long-Term Upper-Limb Impairment after Stroke.** N Engl J Med 2010, **362**(19):1772-1783.

88. Lorenz MW, Polak JF, Kavousi M, Mathiesen EB, Völzke H, Tuomainen T-, Sander D, Plichart M, Catapano AL, Robertson CM, Kiechl S, Rundek T, Desvarieux M, Lind L, Schmid C, DasMahapatra P, Gao L, Ziegelbauer K, Bots ML, Thompson SG: **Carotid intima-media thickness progression to predict cardiovascular events in the general population (the PROG-IMT collaborative project): A meta-analysis of individual participant data.** The Lancet 2012, **379**(9831):2053-2062.

89. Lu C, Schneider MT, Gubbins P, Leach-Kemon K, Jamison D, Murray CJ: **Public financing of health in developing countries: a cross-national systematic analysis.** The Lancet 2010, **375**(9723):1375-1387.

90. Marcus CL, Moore RH, Rosen CL, Giordani B, Garetz SL, Taylor HG, Mitchell RB, Amin R, Katz ES, Arens R, Paruthi S, Muzumdar H, Gozal D, Thomas NH, Ware J, Beebe D, Snyder K, Elden L, Sprecher RC, Willging P, Jones D, Bent JP, Hoban T, Chervin RD, Ellenberg SS, Redline S: **A Randomized Trial of Adenotonsillectomy for Childhood Sleep Apnea.** N Engl J Med 2013, **368**(25):2366-2376.

91. Martin DF, Maguire MG, Ying G-, Grunwald JE, Fine SL, Jaffe GJ: **Ranibizumab and bevacizumab for neovascular age-related macular degeneration.** N Engl J Med 2011, **364**(20):1897-1908.

92. Mauer M, Zinman B, Gardiner R, Suissa S, Sinaiko A, Strand T, Drummond K, Donnelly S, Goodyer P, Gubler MC, Klein R: **Renal and Retinal Effects of Enalapril and Losartan in Type 1 Diabetes.** N Engl J Med 2009, **361**(1):40-51.

93. May M, Boulle A, Phiri S, Messou E, Myer L, Wood R, Keiser O, Sterne JA, Dabis F, Egger M: **Prognosis of patients with HIV-1 infection starting antiretroviral therapy in sub-Saharan Africa: A collaborative analysis of scale-up programmes.** The Lancet 2010, **376**(9739):449-457.

94. McManus RJ, Mant J, Bray EP, Holder R, Jones MI, Greenfield S, Kaambwa B, Banting M, Bryan S, Little P, Williams B, Hobbs FR: **Telemonitoring and self-management in the control of hypertension (TASMINH2): a randomised controlled trial.** The Lancet 2010, **376**(9736):163-172.

95. Melhuish E, Belsky J, Leyland AH, Barnes J: **Effects of fully-established Sure Start Local Programmes on 3-year-old children and their families living in England: a quasi-experimental observational study.** The Lancet 2008, **372**(9650):1641-1647.

96. Milstone AM, Elward A, Song X, Zerr DM, Orscheln R, Speck K, Obeng D, Reich NG, Coffin SE, Perl TM: **Daily chlorhexidine bathing to reduce bacteraemia in critically ill children: a multicentre, cluster-randomised, crossover trial.** The Lancet **381**(9872):1099-1106.

97. Montalescot G, Zeymer U, Silvain J, Boulanger B, Cohen M, Goldstein P, Ecollan P, Combes X, Huber K, Pollack Jr C, Bénezet J, Stibbe O, Filippi E, Teiger E, Cayla G, Elhadad S, Adnet F, Chouihed T, Gallula S, Greffet A, Aout M, Collet J, Vicaut E: **Intravenous enoxaparin or unfractionated heparin in primary percutaneous coronary intervention for ST-elevation myocardial infarction: the international randomised open-label ATOLL trial.** The Lancet 2011, **378**(9792):693-703.

98. Mozaffarian D, Shi P, Morris JS, Spiegelman D, Grandjean P, Siscovick DS, Willett WC, Rimm EB: **Mercury Exposure and Risk of Cardiovascular Disease in Two U.S. Cohorts.** N Engl J Med 2011, **364**(12):1116-1125.

99. Odd DE, Lewis G, Whitelaw A, Gunnell D: **Resuscitation at birth and cognition at 8 years of age: a cohort study.** The Lancet 2009, **373**(9675):1615-1622.

100. Olanow CW, Rascol O, Hauser R, Feigin PD, Jankovic J, Lang A, Langston W, Melamed E, Poewe W, Stocchi F, Tolosa E: **A Double-Blind, Delayed-Start Trial of Rasagiline in Parkinson's Disease.** N Engl J Med 2009, **361**(13):1268-1278.

101. Pandharipande PP, Girard TD, Jackson JC, Morandi A, Thompson JL, Pun BT, Brummel NE, Hughes CG, Vasilevskis EE, Shintani AK, Moons KG, Geevarghese SK, Canonico A, Hopkins RO, Bernard GR, Dittus RS, Ely EW: **Long-Term Cognitive Impairment after Critical Illness.** N Engl J Med 2013, **369**(14):1306-1316.

102. Parker C, Waters R, Leighton C, Hancock J, Sutton R, Moorman AV, Ancliff P, Morgan M, Masurekar A, Goulden N, Green N, Révész T, Darbyshire P, Love S, Saha V: **Effect of mitoxantrone on outcome of children with first relapse of acute lymphoblastic leukaemia (ALL R3): an open-label randomised trial.** The Lancet 2010, **376**(9757):2009-2017.

103. Patel V, Weiss HA, Chowdhary N, Naik S, Pednekar S, Chatterjee S, De Silva MJ, Bhat B, Araya R, King M, Simon G, Verdeli H, Kirkwood BR: **Effectiveness of an intervention led by lay health counsellors for depressive and anxiety disorders in primary care in Goa, India (MANAS): a cluster randomised controlled trial.** The Lancet 2010, **376**(9758):2086-2095.

104. Peek M, Mugford M, Tiruvoipati R, Wilson A, Allen E, Thalanany MM, Hibbert CL, Truesdale A, Clemens F, Cooper N, Firmin RK, Elbourne D: **Efficacy and economic assessment of conventional ventilatory support versus extracorporeal membrane oxygenation for severe adult respiratory failure (CESAR): a multicentre randomised controlled trial.** The Lancet 2009, **374**(9698):1351-1363.

105. Pimentel M, Lembo A, Chey WD, Zakko S, Ringel Y, Yu J, Mareya SM, Shaw AL, Bortey E, Forbes WP: **Rifaximin Therapy for Patients with Irritable Bowel Syndrome without Constipation.** N Engl J Med 2011, **364**(1):22-32.

106. Price D, Musgrave SD, Shepstone L, Hillyer EV, Sims EJ, Gilbert RFT, Juniper EF, Ayres JG, Kemp L, Blyth A, Wilson ECF, Wolfe S, Freeman D, Mugford HM, Murdoch J, Harvey I: **Leukotriene Antagonists as First-Line or Add-on Asthma-Controller Therapy.** N Engl J Med 2011, **364**(18):1695-1707.

107. Pronyk PM, Muniz M, Nemser B, Somers M-, McClellan L, Palm CA, Huynh UK, Amor YB, Begashaw B, McArthur JW, Niang A, Sachs SE, Singh P, Teklehaimanot A, Sachs JD: **The effect of an integrated multisector model for achieving the Millennium Development Goals and improving child survival in rural sub-Saharan Africa: A non-randomised controlled assessment.** The Lancet 2012, **379**(9832):2179-2188.

108. Puhan MA, Garcia-Aymerich J, Frey M, ter Riet G, Antó JM, Agustí AG, Gómez FP, Rodríguez-Roisín R, Moons KG, Kessels AG, Held U: **Expansion of the prognostic assessment of patients with chronic obstructive pulmonary disease: the updated BODE index and the ADO index.** The Lancet **374**(9691):704-711.

109. Roberts JD, Wells GA, Le May MR, Labinaz M, Glover C, Froeschl M, Dick A, Marquis J, O'Brien E, Goncalves S, Druce I, Stewart A, Gollob MH, So DY: **Point-of-care genetic testing for personalisation of antiplatelet treatment (RAPID GENE): a prospective, randomised, proof-of-concept trial.** The Lancet 2012, **379**(9827):1705-1711.

110. Sitas F, Egger S, Bradshaw D, Groenewald P, Laubscher R, Kielkowski D, Peto R: **Differences among the coloured, white, black, and other South African populations in smoking-attributed mortality at ages 35–74 years: a case-control study of 481 640 deaths.** The Lancet 2013, **382**(9893):685-693.

111. Smith KR, McCracken JP, Weber MW, Hubbard A, Jenny A, Thompson LM, Balmes J, Diaz A, Arana B, Bruce N: **Effect of reduction in household air pollution on childhood pneumonia in Guatemala (RESPIRE): A randomised controlled trial.** The Lancet 2011, **378**(9804):1717-1726.

112. Solomon SD, Zile M, Pieske B, Voors A, Shah A, Kraigher-Krainer E, Shi V, Bransford T, Takeuchi M, Gong J, Lefkowitz M, Packer M, McMurray JJ: **The angiotensin receptor neprilysin inhibitor LCZ696 in heart failure with preserved ejection fraction: a phase 2 double-blind randomised controlled trial.** The Lancet 2012, **380**(9851):1387-1395.

113. Sommers BD, Baicker K, Epstein AM: **Mortality and access to care among adults after state medicaid expansions.** N Engl J Med 2012, **367**(11):1025-1034.

114. Stanworth SJ, Estcourt LJ, Powter G, Kahan BC, Dyer C, Choo L, Bakrania L, Llewelyn C, Littlewood T, Soutar R, Norfolk D, Copplestone A, Smith N, Kerr P, Jones G, Raj K, Westerman DA, Szer J, Jackson N, Bardy PG, Plews D, Lyons S, Bielby L, Wood EM, Murphy MF: **A No-Prophylaxis Platelet-Transfusion Strategy for Hematologic Cancers.** N Engl J Med 2013, **368**(19):1771-1780.

115. Sterne J, May M, Costagliola D, de Wolf F, Phillips AN, Harris R, Funk MJ, Geskus RB, Gill J, Dabis F, Miro JM, Justice AC, Ledergerber B, Fatkenheuer G, Hogg RS, Monforte AD, Saag M, Smith C, Staszewski S, Egger M, Cole SR: **Timing of initiation of antiretroviral therapy in AIDS-free HIV-1-infected patients: a collaborative analysis of 18 HIV cohort studies.** *Lancet* 2009, **373**(9672)**:**1352-1363.

116. Strang J, Metrebian N, Lintzeris N, Potts L, Carnwath T, Mayet S, Williams H, Zador D, Evers R, Groshkova T, Charles V, Martin A, Forzisi L: **Supervised injectable heroin or injectable methadone versus optimised oral methadone as treatment for chronic heroin addicts in England after persistent failure in orthodox treatment (RIOTT): a randomised trial.** The Lancet 2010, **375**(9729):1885-1895.

117. Subak LL, Wing R, West DS, Franklin F, Vittinghoff E, Creasman JM, Richter HE, Myers D, Burgio KL, Gorin AA, Macer J, Kusek JW, Grady D: **Weight Loss to Treat Urinary Incontinence in Overweight and Obese Women.** N Engl J Med 2009, **360**(5):481-490.

118. Tanser F, Bärnighausen T, Hund L, Garnett GP, McGrath N, Newell M-: **Effect of concurrent sexual partnerships on rate of new HIV infections in a high-prevalence, rural South African population: A cohort study.** The Lancet 2011, **378**(9787):247-255.

119. Thabut G, Christie JD, Ravaud P, Castier Y, Brugière O, Fournier M, Mal H, Lesèche G, Porcher R: **Survival after bilateral versus single lung transplantation for patients with chronic obstructive pulmonary disease: a retrospective analysis of registry data.** The Lancet 2008, **371**(9614):744-751.

120. Underwood M, Lamb SE, Eldridge S, Sheehan B, Slowther A, Spencer A, Thorogood M, Atherton N, Bremner SA, Devine A, Diaz-Ordaz K, Ellard DR, Potter R, Spanjers K, Taylor SJC: **Exercise for depression in elderly residents of care homes: a cluster-randomised controlled trial.** Lancet 2013, **382**(9886):41-49.

121. Vaucher YE, Peralta-Carcelen M, Finer NN, Carlo WA, Gantz MG, Walsh MC, Laptook AR, Yoder BA, Faix RG, Das A, Schibler K, Rich W, Newman NS, Vohr BR, Yolton K, Heyne RJ, Wilson-Costello DE, Evans PW, Goldstein RF, Acarregui MJ, Adams-Chapman I, Pappas A, Hintz SR, Poindexter B, Dusick AM, McGowan EC, Ehrenkranz RA, Bodnar A, Bauer CR, Fuller J, O'Shea TM, Myers GJ, Higgins RD: **Neurodevelopmental outcomes in the early CPAP and pulse oximetry trial.** N Engl J Med 2012, **367**(26):2495-2504.

122. Visco AG, Brubaker L, Richter HE, Nygaard I, Paraiso MFR, Menefee SA, Schaffer J, Lowder J, Khandwala S, Sirls L, Spino C, Nolen TL, Wallace D, Meikle SF: **Anticholinergic Therapy vs. OnabotulinumtoxinA for Urgency Urinary Incontinence.** N Engl J Med 2012, **367**(19):1803-1813.

123. Wadden TA, Volger S, Sarwer DB, Vetter ML, Tsai AG, Berkowitz RI, Kumanyika S, Schmitz KH, Diewald LK, Barg R, Chittams J, Moore RH: **A Two-Year Randomized Trial of Obesity Treatment in Primary Care Practice.** N Engl J Med 2011, **365**(21):1969-1979.

124. Walkup JT, Albano AM, Piacentini J, Birmaher B, Compton SN, Sherrill JT, Ginsburg GS, Rynn MA, McCracken J, Waslick B, Iyengar S, March JS, Kendall PC: **Cognitive Behavioral Therapy, Sertraline, or a Combination in Childhood Anxiety.** N Engl J Med 2008, **359**(26):2753-2766.

125. Walz G, Budde K, Mannaa M, Nürnberger J, Wanner C, Sommerer C, Kunzendorf U, Banas B, Hörl WH, Obermüller N, Arns W, Pavenstädt H, Gaedeke J, Büchert M, May C, Gschaidmeier H, Kramer S, Eckardt K: **Everolimus in Patients with Autosomal Dominant Polycystic Kidney Disease.** N Engl J Med 2010, **363**(9):830-840.

126. Wang WC, Ware RE, Miller ST, Iyer RV, Casella JF, Minniti CP, Rana S, Thornburg CD, Rogers ZR, Kalpatthi RV, Barredo JC, Brown RC, Sarnaik SA, Howard TH, Wynn LW, Kutlar A, Armstrong FD, Files BA, Goldsmith JC, Waclawiw MA, Huang X, Thompson BW: **Hydroxycarbamide in very young children with sickle-cell anaemia: a multicentre, randomised, controlled trial (BABY HUG).** The Lancet 2011, **377**(9778):1663-1672.

127. Weeks JC, Catalano PJ, Cronin A, Finkelman MD, Mack JW, Keating NL, Schrag D: **Patients' expectations about effects of chemotherapy for advanced cancer.** N Engl J Med 2012, **367**(17):1616-1625.

128. Weintraub WS, Spertus JA, Kolm P, Maron DJ, Zhang Z, Jurkovitz C, Zhang W, Hartigan PM, Lewis C, Veledar E, Bowen J, Dunbar SB, Deaton C, Kaufman S, O'Rourke RA, Goeree R, Barnett PG, Teo KK, Boden WE: **Effect of PCI on Quality of Life in Patients with Stable Coronary Disease.** N Engl J Med 2008, **359**(7):677-687.

129. Weiser TG, Regenbogen SE, Thompson KD, Haynes AB, Lipsitz SR, Berry WR, Gawande AA: **An estimation of the global volume of surgery: a modelling strategy based on available data.** The Lancet 2008, **372**(9633):139-144.

130. Westerlund H, Kivimäki M, Singh-Manoux A, Melchior M, Ferrie JE, Pentti J, Jokela M, Leineweber C, Goldberg M, Zins M, Vahtera J: **Self-rated health before and after retirement in France (GAZEL): a cohort study.** The Lancet 2009, **374**(9705):1889-1896.

131. Wherrett DK, Bundy B, Becker DJ, DiMeglio LA, Gitelman SE, Goland R, Gottlieb PA, Greenbaum CJ, Herold KC, Marks JB, Monzavi R, Moran A, Orban T, Palmer JP, Raskin P, Rodriguez H, Schatz D, Wilson DM, Krischer JP, Skyler JS: **Antigen-based therapy with glutamic acid decarboxylase (GAD) vaccine in patients with recent-onset type 1 diabetes: a randomised double-blind trial.** The Lancet 2011, **378**(9788):319-327.

132. Wiles N, Thomas L, Abel A, Ridgway N, Turner N, Campbell J, Garland A, Hollinghurst S, Jerrom B, Kessler D, Kuyken W, Morrison J, Turner K, Williams C, Peters T, Lewis G: **Cognitive behavioural therapy as an adjunct to pharmacotherapy for primary care based patients with treatment resistant depression: results of the CoBalT randomised controlled trial.** The Lancet 2013, **381**(9864):375-384.

133. Yu H, Cowling BJ, Feng L, Lau EH, Liao Q, Tsang TK, Peng Z, Wu P, Liu F, Fang VJ, Zhang H, Li M, Zeng L, Xu Z, Li Z, Luo H, Li Q, Feng Z, Cao B, Yang W, Wu JT, Wang Y, Leung GM: **Human infection with avian influenza A H7N9 virus: an assessment of clinical severity.** The Lancet 2013, **382**(9887):138-145.

134. Zeig-Owens R, Webber MP, Hall CB, Schwartz T, Jaber N, Weakley J, Rohan TE, Cohen HW, Derman O, Aldrich TK, Kelly K, Prezant DJ: **Early assessment of cancer outcomes in New York City firefighters after the 9/11 attacks: an observational cohort study.** The Lancet 2011, **378**(9794):898-905.
